# On Puerperal Convulsions Prior to Labor

**Published:** 1855-11

**Authors:** 


On Puerperal Convulsions prior to Labor.—During some recent clinical remarks, M. Dubois observed that it was formerly generally believed, and is so still by many, that in these cases we should induce premature labor. He does not agree to this view, and thinks the practice may even be injurious. It is indeed useless, because labor always comes on of itself, under the influence of convulsion, and it is injurious by reason of its irritating the uterus and thus aggravating the convulsions. For the same reasons he objects to the use of ergot. He employs general bleeding, as far as the strength will permit it, leeches behind the ears, and purgatives. Of these last, calomel and jalap, mixed up with honey, and laid on the tongue every hour till it operates, is the best. Sinapisms should also be applied to the feet, and ice to the head. The only operation justifiable is the application of the forceps, when the head is engaged in the pelvis, and solely in aid of the last expulsive efforts.—(*Gaz. des Hôp.*) *Ibid.*

